# Oral lichen planus: A retrospective study of 633 patients from Bucharest, Romania

**DOI:** 10.4317/medoral.18035

**Published:** 2012-12-10

**Authors:** Serban Tovaru, Ioanina Parlatescu, Carmen Gheorghe, Mihaela Tovaru, Mariana Costache, Andrea Sardella

**Affiliations:** 1 Department of Oral Medicine, Faculty of Dental Medicine, University of Medicine and Pharmacy Carol Davila, Bucharest; 2 Department of Dermatology, Faculty of Dental Medicine, University of Medicine and Pharmacy Carol Davila, Bucharest; 3 Department of Pathology, Faculty of Medicine, University of Medicine and Pharmacy Carol Davila, Bucharest; 4 Department of Medicine, Surgery and Dentistry, Unit of Oral Medicine, Oral Pathology and Gerodontology, University of Milan, Milan

## Abstract

Objective: In this retrospective study, patients’ medical records were reviewed to investigate the profiles of 633 OLP cases in a group of Romania. Material and Methods: In this retrospective study, the following clinical data were obtained from the medical charts of patients: gender, age, clinical presentation of OLP, site affected, presence of symptoms, extraoral manifestations of lichen planus, presence of systemic diseases, and history of medications. Results: Most (78.67%) OLP patients were female and the mean age at presentation was 52 years. The white type of the disease (reticular/papular/plaque lesions) was the main form encountered in this sample (48.97%). Among patients with available hepatitis C virus test results, 9.6% were serum-positive. OLP was associated with gallbladder disease (i.e. cholecystitis, cholelithiasis) in 19% of patients. Six patients (0.95%) developed squamous cell carcinoma at a site with confirmed OLP lesions. Conclusions: To the best of our knowledge, no similar study has been conducted in a Romanian population. The present investigation revealed the predominance of OLP among middle-aged white women and the prevalence of bilateral involvement of the buccal mucosa with reticular white lesions. Anti-HCV circulating antibodies were more common in patients with OLP than in the general population and, notably, OLP was associated with gallbladder disease (cholecystitis, cholelithiasis) in 19% of patients.

** Key words:**Oral lichen planus, oral mucosal diseases, retrospective study.

## Introduction

Oral lichen planus (OLP) is a chronic inflammatory disease that affects stratified squamous epithelium. Several immunological mechanisms of its pathogenesis have been proposed, including antigen-specific cell-mediated immune response, nonspecific immunological mechanisms, autoimmune response, and humoural immunity (1). The estimated prevalence of the disease in the general population is 1–1.5% (2), with a predominance among females in the fifth and sixth decades of life. About 15–20% of patients with OLP have or develop skin lesions (3), typically manifesting as erythematous and itchy flat papules on the flexor surfaces of the forearms. Other mucosal sites may be involved, including the genitalia, oesophagus, larynx, and conjunctiva (4). The clinical features of OLP are generally polymorphic and usually consist of bilateral and/or multiple symmetrical lesions, such as white and raised papules or plaques, erosions, or often-painful atrophic lesions (4). Although evidence for the necessity of histological evaluation is not definitive and inter- and intra-observer variability are often high (5), on biopsy OLP shows basal layer destruction and basal membrane interruption due to hydropic degeneration, the apoptosis-related formation of Civatte bodies, and a juxta-epithelial lymphocyte inflammatory infiltrate with a band arrangement (6). OLP has been associated with a risk of malignant transformation that has ranged from 0.4–5% over 0.5–20-year periods of observation (7).

Several epidemiological studies in various parts of the world have described the clinical characteristics of OLP. However, few studies have examined OLP in Eastern Europe. The purpose of this study was to describe the clinical characteristics and associated systemic diseases of OLP in 633 Romanian patients.

## Material and Methods

An observational and retrospective study was conducted at the Department of Oral Medicine and Oral Pathology, Faculty of Dental Medicine, Carol Davila University, Bucharest, Romania. A total of 889 medical charts of patients diagnosed with OLP between January 1990 and September 2010 were analysed by two experienced oral physicians (ST, AS) and an experienced oral pathologist (MC). The Bioethics Committee of the University Carol Davila was informed about the retrospective evaluation process on medical charts. The following clinical data were obtained from the medical charts: gender, age, clinical presentation of OLP, site affected, presence of symptoms, extraoral manifestations of lichen planus, presence of systemic diseases, and history of medications. Major stressful events (e.g. death of family member, divorce, job loss, major accident) were taken into account. Records of patients diagnosed with lichenoid lesions were excluded from the sample. In particular, we excluded patients with oral lichenoid contact lesions (OLCL) resulting from allergic contact stomatitis (delayed immune mediated hypersensitivity), most commonly in direct topographic relationship to dental restorative materials (5), and patients with oral lichenoid drug reactions (OLDR), which arise in temporal association with the use of some medications (e.g. oral hypoglycaemic agents, angiotensin-converting enzyme inhibitors) (8).

For the clinical classification, we used the modified WHO criteria proposed by van der Meij and van der Waal (9). Only pa-tients with clinical and histological evidence of OLP were included in the study. Incomplete or inaccurate records (256/889, 32%) were not considered. The final sample thus consisted of 633 records.

## Results

Data were obtained from the medical records of 633 patients with clinically and histopathologically confirmed diagnoses of OLP. Most (498/633, 78.67%) OLP patients were female. The mean age at presentation was 52 years among women and 64 years among men. The majority (52%) of patients were referred by general dentists. General medical practitioners (family physicians) referred 24% of the OLP patients and nearly 21% of patients sought consultation spontaneously.

**Figure 1:**
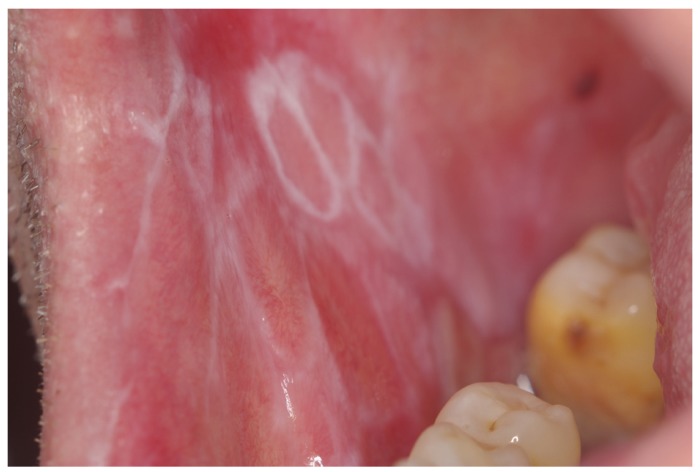
Reticular white lesions on the left buccal mucosa of a male patient.

**Figure 2:**
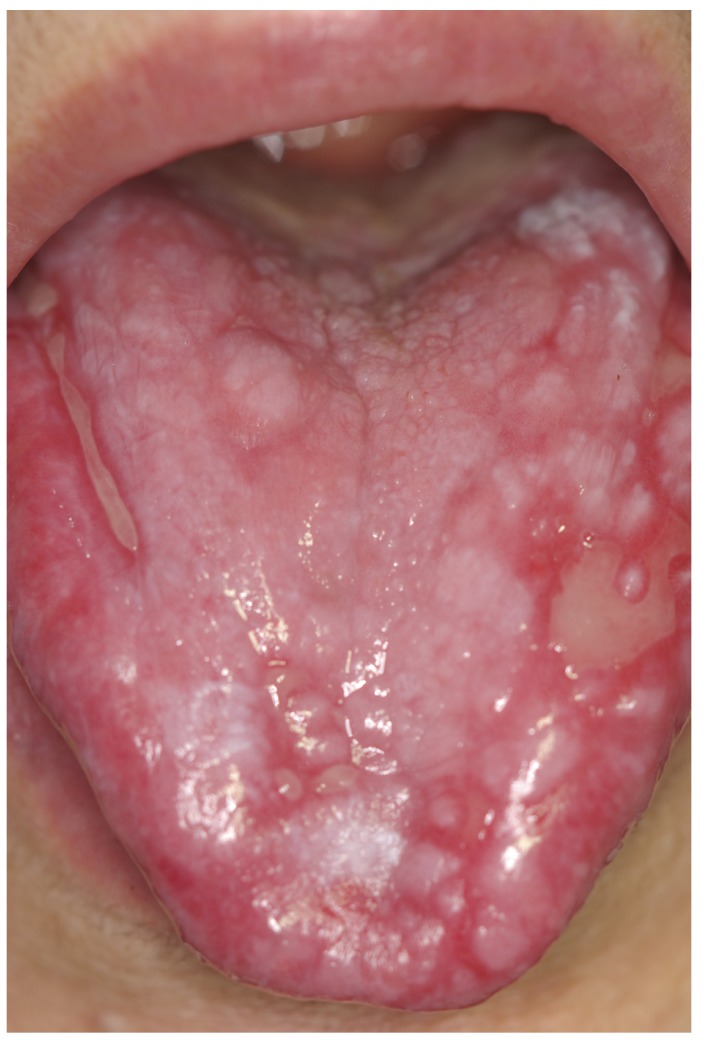
Lingual involvement of the same patient of Figure 2.

The white type of the disease (reticular/papular/plaque lesions) was the main form encountered in this sample (310/633, 48.97%), (Figs. 1,2). The ulcerative form of OLP was observed in 35.86% of patients (227/633). Atrophic-erosive lesions were observed in 13.59% of patients (86/633), sometimes concomitantly with reticular lesions. The bullous form of OLP was observed in only 10 patients ( Table 1).

**Table 1:**
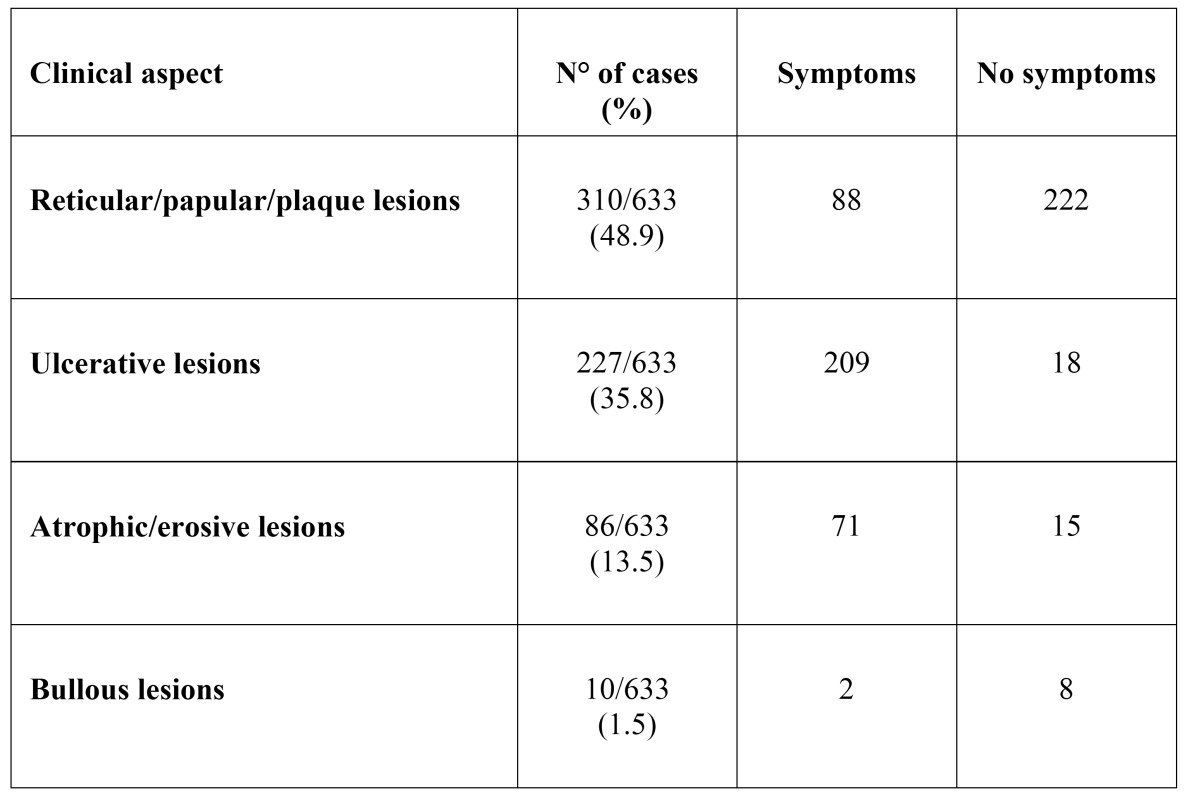
Clinical presentation and symptoms in 633 OLP patients.

Among all 633 patients, the buccal mucosa was the most common site of involvement, followed by the tongue, gingiva, labial mucosa, and floor of the mouth. Lesions on the palate were observed rarely.

The majority (58%) of patients reported at least one symptom, and atrophic-erosive lesions and ulcerative forms were associated with significantly more symptom-related complaints (e.g. pain, burning sensation, bleeding), ( Table 1).

Almost 25% of patients had skin involvement, as confirmed by a senior dermatologist who evaluated the medical records (MT). In particular, 130 patients showed the typical skin lesions (shiny, violaceous, flat-topped, polygonal papules, sometimes with fine white Wickham’s striae). Few patients had scalp or nail involvement (2% of patients). Again, lesions of the oral mucosa were rarely associated with those of the genital mucosa (2% of patients). In 3 patients only were reported oesophageal involvement.

Major stress (e.g. death in family, divorce, job loss, major accident) was a factor in 26.26% (151/633) of patients.

Liver profiles including serum transaminase levels, total bilirubin, antibodies to hepatitis B and C viruses (the latter available regularly only after 1995, obtained using ELISA with standardised reagents), and hepatitis B surface antigen showed that 24% (179/633) of patients were affected by liver abnormalities. In particular, 9.64% (61/456) of patients with available hepatitis C virus (HCV) test results were HCV serum-positive.

OLP was associated with gallbladder diseases in 19% (121/633) of patients. Such diseases consisted mainly of cholecystitis, cholelithiasis (gallstones), and abscesses, with a few cases of sclerosing cholangitis and tumours. Diabetes mellitus, mainly type 2, affected 10% of OLP patients.

Six patients (0.95%) developed squamous cell carcinoma at a site with confirmed OLP lesions ( Table 2). All but one malignant transformation occurred in erosive or erythematous types of lichen planus, with no dysplasia reported in initial biopsies. One malignant transformation arose in a reticular form of OLP.

**Table 2:**
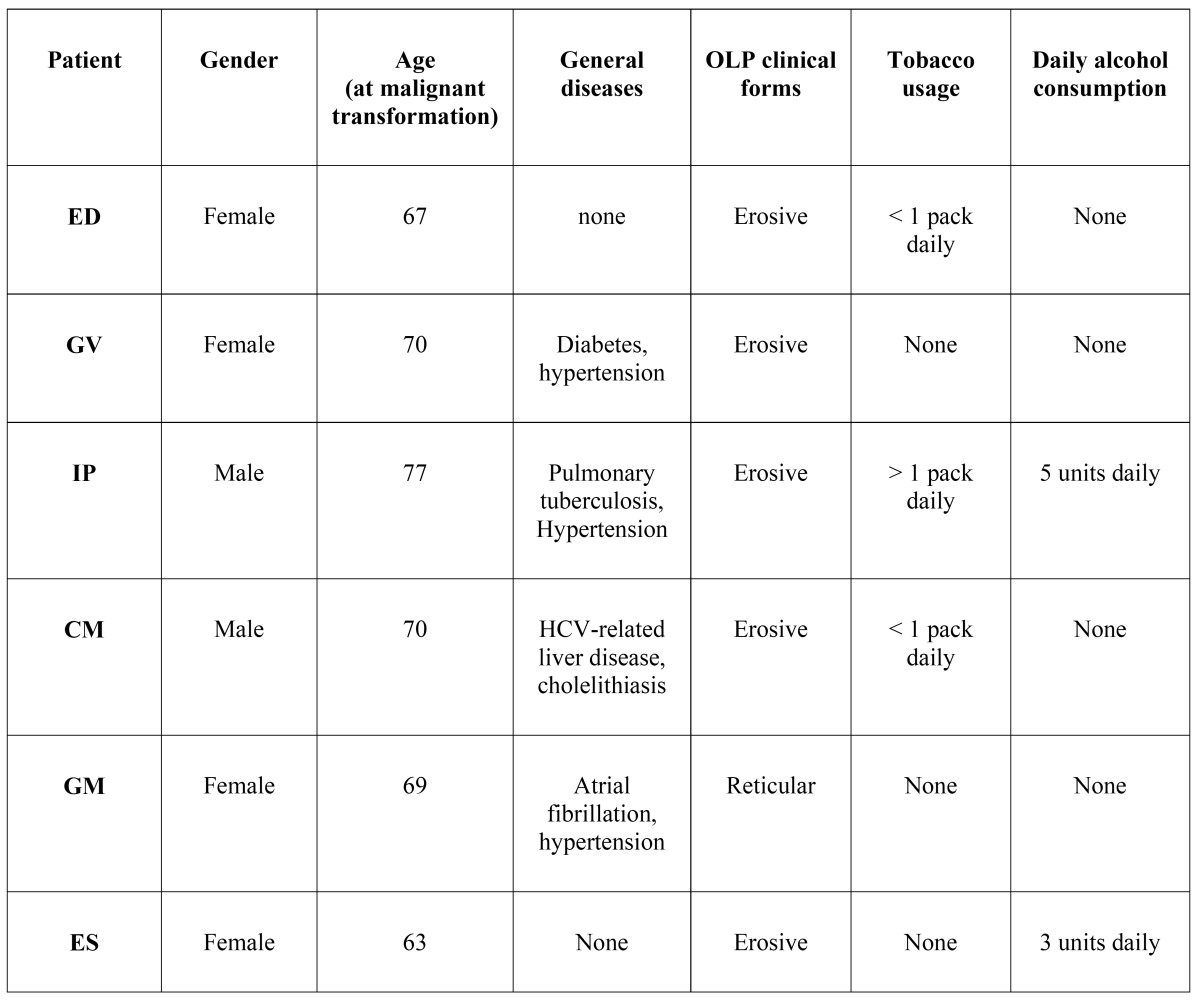
Characteristics of OLP patients with malignant development.

## Discussion

The clinical characteristics of the patients studied in this cohort were similar to those reported in the literature. The predominance of OLP in female patients and in those in the fifth through seventh decades of life was observed in the present study, in agreement with other reports (10-12).

The buccal mucosa was the most common site of OLP lesions in our sample, which is similar to the findings of previous reports (10-13).

The white type of OLP (reticular/papular/plaque lesions) was predominant in this Romanian sample (48.97% of 633 patients), in agreement with three reports from Croatia that found white forms of OLP in 66.9% (14), 88.5% (15), and 62% (16) of patients considered. Similar results were also observed in an Iranian sample (77% keratotic forms), (17) although the authors found a higher prevalence of OLP in the third to fourth decades of life (mean age: 41.6 years). This age distribution differs from that in our Romanian patients, in which OLP occurred at a mean age of 52 years in women and 64 years in men.

In a study of Croatian patients (14), OLP was found to be associated most commonly with hepatobiliary diseases (cholecystopa-thy, cholelithiasis, increased bilirubin value). Liver disease (including hepatitis and cirrhosis) was also detected in a group of Croatian OLP patients (42/175, 24%), (16). In our sample, 19% of OLP patients had gallbladder diseases, represented mainly by cholecystitis and cholelithiasis (gallstones). Compared with the general Romanian population, OLP patients had a higher preva-lence of gallbladder diseases (19% vs. 8.4%), (18).

In our sample, 9.6% of patients were HCV serum-po-sitive. Epidemiological data suggest that LP may be associated significantly with HCV infection in Southern Europe and Japan, but not in northern Europe. This geographical difference may be due to immunogenetic factors, duration of HCV infection, and differences in study design (19). A recent nationwide cross-sectional survey (20) conducted from 2006–2008 using population-stratified, random cluster sampling found HCV infection in 3.23% of the Romanian adult population. Anti-HCV circulating antibodies thus appear to be more common in patients with OLP than in the general population.

Diabetes mellitus, mainly type 2, affected 10% of the OLP patients in our sample, reflecting the prevalence of diabetes (types 1 and 2) in the general population of Romania (8–9%), (21).

OLP is considered to be a potentially malignant disorder of the oral mucosa (22). The most important complication of this disease may be the development of oral squamous carcinoma, although this is a very controversial topic (23). In our sample, six patients (0.95%) developed squamous cell carcinoma at a site with confirmed OLP lesions. Five malignant transformations occurred in erosive or atrophic types of lichen planus, with no dysplasia noted in initial biopsies, and one malignant transformation arose in a reticular form of OLP. This percentage is rather similar to other reported data. A study in south-eastern Spain (13) found a malignization rate of 0.90% (8/550 OLP patients). Carbone et al. (24) reported that 1.85% of 808 OLP patients in Italy developed oral carcinoma during the follow-up period. Thirteen of 690 (1.9%) British OLP patients (11) developed oral squamous carcinoma and four of 141 (2.8%) patients in Switzerland (25) showed oral carcinoma at a site with OLP lesions. Given that chronic inflammation has been associated causally with various types of cancer, the malignant transformation of OLP may be related to, or dependent on, a series of molecular stimuli originating in the inflammatory infiltrate (e.g. cytokine and chemokine release by infiltrating T cells). These stimuli may induce fundamental protein changes in oral epithelial cells, leading to the progression of OLP to oral squamous cell carcinoma (26).

In this cohort, 26% of patients reported an exacerbation of OLP symptoms in periods of greater emotional tension and/or anxiety. In a recent paper, Manolache and colleagues (27) evaluated the possible role of stress in the onset/extension of cutaneous lichen planus in patients treated at the dermatological department of Cetatea Histria Polyclinic in Bucharest, Romania. In this case-control study, the authors identified at least one potentially stressful situation in 31 cases (67.39%), compared with the occurrence of such situations in 10 control patients (21.73%). Psychological disturbances have been investigated in the etiopathogenesis of OLP. Stress, as well as other psychological alterations, seems to modify and promote dysregulation of immune functions by altering the Th1/Th2 cytokine balance and increasing Th2 response, which is associated with the development of autoimmune diseases (28). Although some investigations (29,30) have failed to replicate the association between OLP and stressful events, psychological/psychiatric services should be combined with conventional therapy for these patients to avoid the occurrence of somatisation and to prevent disease exacerbation.

Although observational retrospective studies have various limitations, to the best of our knowledge, no similar study has been conducted in a Romanian population. The present investigation revealed the predominance of OLP among middle-aged white women and the prevalence of bilateral involvement of the buccal mucosa. Reticular lesions were most frequent, followed by the erosive form, which is most frequently associated with painful symptoms. Anti-HCV circulating antibodies were more common in patients with OLP than in the general population and, notably, OLP was associated with gallbladder disease (cholecystitis, cholelithiasis) in 19% of patients.
